# Habits and Attitudes of Video Gaming and Information Technology Use in People with Schizophrenia: Cross-Sectional Survey

**DOI:** 10.2196/14865

**Published:** 2020-07-22

**Authors:** William TH Choi, Dan KS Yu, Terry Wong, Tella Lantta, Min Yang, Maritta Välimäki

**Affiliations:** 1 School of Nursing The Hong Kong Polytechnic University Hong Kong Hong Kong; 2 The Mental Health Association of Hong Kong Hong Kong Hong Kong; 3 New Life Psychiatric Rehabilitation Association Hong Kong Hong Kong; 4 Department of Nursing Science, Faculty of Medicine University of Turku Turku Finland; 5 West China School of Public Health Sichuan University Huaxi Medical Center Sichuan China; 6 Faculty of Health, Design and Art Swinburne University of Technology Melbourne Australia

**Keywords:** video gaming, internet, information technology, schizophrenia

## Abstract

**Background:**

Information technology and video gaming have potential advantages in the treatment of schizophrenia. However, information regarding the habits and attitudes related to internet use and video gaming in people with schizophrenia is limited.

**Objective:**

The aim of this study was to explore the habits and attitudes regarding video gaming and information technology usage and their associated factors in people with schizophrenia in Hong Kong.

**Methods:**

In this cross-sectional survey, service users with schizophrenia were recruited from 6 halfway hostels and 7 integrated centers for mental wellness in Hong Kong. A 79-item self-report questionnaire was utilized to explore the habits of internet use and video gaming in these people with schizophrenia. The attitude toward video gaming was assessed using the Gaming Attitudes, Motivations, and Experiences Scales. Of the 148 individuals in a convenience sample who were invited to participate in this study, 110 willingly participated (a response rate of 74.3%). The data were analyzed using descriptive statistics, a two-tailed independent t test, Pearson correlation, and principal analysis with 3 methods of rotation (varimax, equimax, and promax).

**Results:**

Most participants (100/110, 90.9%) had access to the internet and half of them (54/110, 49.1%) used the internet daily mostly to watch videos (66/110, 60.0%) or read news or books, etc (42/110, 38.2%). One-third of the participants (36/110, 32.7%) used the internet to play web-based games, and most of them (88/110, 80.0%) had played a video game in the past year. The most favorable gaming platforms were cellular phones (43/88, 49%) followed by computers (19/88, 22%) and arcade cabinets (6/88, 7%). The most favorable game genre was action games (34/145, 23.4%). Those who had a bachelor’s degree or higher scored lower in social interaction than those with a lower education level (*P*=.03). Those who played video games daily scored higher in the category of story than those who did not play daily (t_86_=2.03, *P*=.05). The most popular gaming category was autonomy and the least popular categories were violent catharsis and violent reward. Two motives, “social playing” and “evasive playing,” were formed to describe the characteristics of playing video games.

**Conclusions:**

Our data showed a high internet utilization rate among people with schizophrenia in Hong Kong. Only a few of them used the internet to search for health-related information. Our study also exemplified the unique habits of gaming among the participants. Health care professionals could utilize video games to engage people with schizophrenia and promote coping with stress and provide social skills training to such people with schizophrenia. Identification of the gaming attitudes can contribute to the development of serious games for the schizophrenic population. Further investigation is vital for the promotion of mental health through web-based platforms.

## Introduction

Schizophrenia is a serious mental illness that is associated with significant cognition and functioning disabilities [1,2]. According to the World Health Organization [3], there are more than 21 million people diagnosed with schizophrenia worldwide. Many of them live in isolation [4,5] and are lonely without social interactions [3,6,7]. A recent systematic review investigated the situation of schizophrenic patients in 24 countries and revealed that the annual cost for the population of patients with schizophrenia ranged from US $94 billion to US $102 billion per country, which consumes up to 1.65% of a country’s gross domestic product [8]. These expenses cover antipsychotic treatments and long-term psychological interventions [9], which are the recommended treatments for schizophrenia [10], and these treatments have demonstrated optimal control over positive symptoms. However, multiple factors hinder the efficacy of this treatment, including the patients’ limited insight into mental illness [11], noncompliance [12], and treatment resistance [13,14]. The management of negative symptoms also remains a clinical challenge [15]. Therefore, new types of treatment methods are needed to promote recovery and optimize rehabilitation [15].

In recent years, different types of video games, including computer games [16,17], mobile games [18], serious games [19-23], and virtual reality [24-26], have been created as adjunctive treatments for various illnesses and they have resulted in desirable outcomes. Scholars suggest that video gaming is a novel intervention for social reconstruction and skills training in people with mental illness [16,19,21,27]. The use of video gaming in psychiatric treatment is promising because video gaming is a rapidly expanding market. In 2018, there were 2.3 billion gamers making up the total revenue of US $137.9 billion globally [28]. The Asia-Pacific region was the largest contributing region, which shared 52% of the worldwide revenue [28]. Mobile gaming has increased by 25.5% per year from 2017 and it now holds 51% of the market. In contrast, a downtrend has been noted for computer gaming [28]. Although the use of information and communication technologies has been rapidly expanding in the world, the accessibility, frequency, and purpose of using information and communication technologies vary among geographical and demographical areas [6,29-32]. In the United States, 97% of the citizens possess a personal computer and 81% have a smartphone, which they use every day [6].

In the United States, 89% of the people with schizophrenia possess a computer and 54% have a smartphone [29]. The majority of these people spend more than 1 hour per day on a computer and a mobile phone, and the main purpose of use includes surfing the internet, social networking, and playing web-based games. One study in Finland revealed that only 55% of the people with schizophrenia had a computer and even fewer had access to internet connection [30]. A recent pilot study investigated the usage of mobile and computer devices among people with severe mental illness [31]. Despite up to 45% of them showing interest in using the devices to support recovery, the majority only used them for general purposes such as listening to music (60%), accessing the internet (59%), and for making calls (59%) [31]. Specifically, young people were more likely to possess a computer and have internet connection, and the main purpose of using the internet included seeking information, studying, web-based chatting, shopping, and gaming [6,30].

People with schizophrenia have positive attitudes toward modern information technology use [33], including gaming [34,35]. Gaming attitudes specifically refers to gamers’ motives and preferences regarding video games [32] and these attitudes vary among gamers with different backgrounds [36]. Ryan et al [36] explored gaming attitudes through the application of the self-determination theory and suggested that if a gamer’s preferences are satisfied, they experience enjoyment when playing the game. Researchers have also identified certain game features to describe the gaming attitudes of gamers, such as storytelling, individual games, and social games [32,36]. Hilgard et al [32] identified 9 factors to measure the motives and preferences of gamers: story, violent catharsis, violent reward, social interaction, escapism, loss aversion, customization, grinding, and autonomy. These factors vary among different players depending on their characteristics such as their social motives, favorite games, and gaming platforms [32]. In general, gaming serves as a primary source of entertainment while simultaneously aiding in constructing and maintaining interpersonal relationships [16]. Beyond enjoyment, players can learn and develop new skills through gaming [16,29]. A study in the United States found that video games are most frequently utilized as a stress reliever; gamers enjoy playing video games as it takes them away from reality. Most video gamers enjoy playing with friends, while male gamers prefer competitions and feel proud when they achieve awards in a game [37].

Recently, gaming has been seen as a possible method of rehabilitation for people with schizophrenia [34]. However, there is still a lack of knowledge about the habits and attitudes of video gaming among people with schizophrenia [15,21,22,24,27,38,39]. Additionally, cultural differences in the attitudes of gaming [40] among people with schizophrenia might exist and have a potential impact on the treatment focus. By exploring the knowledge gap, we might contribute to the development of new interventions for the management of schizophrenia in the future [15]. Therefore, we conducted a survey to explore the habits and attitudes of video gaming and information technology use among people with schizophrenia in Hong Kong and the factors that are associated with their gaming attitudes.

## Methods

### Study Design

A cross-sectional survey design was adopted in this study because it allowed us to explore the attitudes of the respondents by using a structured survey format [41-44].

### Ethical Approval

This study was designed to fulfill the ethical assessments mentioned in the Declaration of Helsinki [45]. This study was assessed by the Human Subjects Ethics subcommittee at the Hong Kong Polytechnic University (HSEARS20180313005) and approved by study setting organizations. An information sheet with the explanation of the study and the rights of participants was provided to each participant. Informed consent was obtained from each participant and witnessed and countersigned by a researcher. The study was guided by the ethical principles of autonomy, confidentiality, anonymity, and nonmalfeasance [45]. It emphasized voluntary participation and participants having the right to withdraw from the study at any time [41,45]. The personal identity of the participants was protected such that their identities were not reported in any publication [45]. Collected data were kept in a locked cabinet and password-protected files, which were only accessible to the research personnel [41]. If there was any concern of emotional burden, participants were free to contact the research team for support through the contact information printed on the information sheet [46].

### Setting

This study was performed in 2 settings, which are both in Hong Kong: integrated community centers for mental wellness and halfway hostels. In Hong Kong, rehabilitation services for people with mental illness in the community are operated by nongovernmental organizations, which operate various services to support the rehabilitation of people with mental illness living in the community such as the settings used in this study. There are 24 integrated community centers for mental wellness providing community support to individuals and their families on a district basis [47] and 36 halfway hostels providing residential support in Hong Kong [48]. Altogether, these types of services cover more than 26,000 citizens annually [49]. An invitation letter was sent to all the nongovernmental organizations that operated integrated community centers for mental wellness and halfway hostels in Hong Kong. Out of the 13 possible nongovernmental organizations, 2 agreed to join the study. Finally, the study was performed at 7 integrated centers for mental wellness and 6 halfway hostels.

### Target Population

The target population consisted of people with schizophrenia who were living in the community [1,3,5]. At the time of the study, there were approximately 48,000 citizens who had been diagnosed with schizophrenia in Hong Kong, and most of them resided in the community [49]. Unlike those who were receiving inpatient psychiatric treatments, people living in the community were free to access video games and information technologies [50]. Therefore, it was more appropriate to explore the habits and gaming attitudes of the people with schizophrenia who resided in the community [27,32,38].

### Recruitment and Sampling

This study adopted convenience sampling in the participant recruitment. Poster promotion and data collection sessions were first arranged at the respective study organizations. Second, inclusion and exclusion criteria for the study participants were screened by representatives of the nongovernmental organizations. This was done to protect the privacy of the participants [45]. The inclusion criteria were as follows: (1) primary diagnosis of schizophrenia (schizophrenia spectrum and other psychotic disorders) under the Diagnostic and Statistical Manual of Mental Disorders, Fifth Edition [1]; (2) aged 18 to 64 years and capable of giving informed consent without a legal guardian; (3) able to read and understand traditional Chinese, which was the prevailing language and understandable by the majority of the citizens in Hong Kong; (4) willing and able to give informed consent [45]; and (5) a resident in the community. The exclusion criteria were as follows: (1) aged <18 or >64 years; (2) meeting diagnostic criteria for a current major depressive, manic, or hypomanic episode (Diagnostic and Statistical Manual of Mental Disorders, Fifth Edition), intellectually disabled, severe visual impairment [1]; (3) active gamer (ie, playing >5 hours/day), for screening purposes only [51]; (4) meeting the diagnostic criteria for substance use disorder (other than tobacco use) [1], head injury, hemiplegia, or other neurological disorders.

### Instruments

#### Background Information of the Participants

Demographic characteristics were collected, including age, gender, education qualification, (no formal education, primary and middle school, high school, college or vocational training, bachelor’s degree, master’s degree, doctoral degree), employment status (unemployed, social welfare benefit, student, disability pension, employed [including sick leave], other), marital status (single, partnership or married, separated or divorced, widowed), residential condition (own household [with partner or family], own household [alone], flat share, parents’ household, supported housing), and the age of the first contact with psychiatric services.

#### Internet Use

The use of the internet was explored using a set of questions extracted from Choi and DiNitto’s study [52]. The questionnaire consisted of 6 items, that is, internet connection (yes/no), possession of email address (yes/no), frequency of using the internet (at least once a day, every few days, once a week, a few times a month, once a month or less often than that), purpose of using the internet (seeking information, communication, shopping, gaming, peer support, others), accessibility to web information (5-point Likert scale, 1=always easy, 5=very difficult), and issues that posed problems in using the internet (problems with limbs, hands, concentration, long-term sitting, eyes, others).

#### Habits of Video Gaming

Habits of video gaming were explored using 7 items, namely, video gaming in the past year (yes/no), seriousness of video gaming (5-point Likert scale, 1=very intense, 5=very casual), frequency of video gaming (daily, 2-3 times per week, weekly, 2-3 times per month, monthly, less than monthly), proportion of spare time for video gaming (5-point Likert scale, 1=almost all of the spare time, 5=almost none of the spare time), favorite and current video games (respondents could list 3 of each), and favorite gaming platform. Subjects were not required to continue answering questions if they indicated that they had not a played a video game within the past year [6,47].

#### Gaming Attitudes

Gaming attitudes were assessed using the Gaming Attitudes, Motivations, and Experiences Scales (GAMES) [32]. The GAMES has 59 items to measure the gaming attitudes, habits, and preferences of the gamers. The items are classified into 9 categories: story (12 items), violent catharsis (7 items), violent reward (6 items), social interaction (6 items), escapism (6 items), loss aversion (7 items), customization (4 items), grinding (6 items), and autonomy (5 items, Table 1) [32]. Each item was rated on a 5-point Likert scale (1=strongly disagree, 5=strongly agree). The summation of the items formed the subscores of the 9 categories; the higher the score reflected the higher preference for a specific category. Factor analysis procedures were conducted with split-halves exploratory factor analysis and confirmatory factor analysis [32]. This instrument possesses good internal consistency in all categories with the Cronbach α=.79-.92 [32].

**Table 1:** GAMES^a^ categories, score range, and their definitions.

Category	Score range	Definition
Story	12-60	Game stories are important, engaging, and emotionally compelling.
Violent catharsis	7-35	Game violence is perceived to harmlessly help release negative moods or aggression.
Violent reward	6-30	Game violence provides positive or thrilling emotions such as satisfaction or power.
Social interaction	6-30	Playing games with a group; developing personal relationships with other players.
Escapism	6-30	Using games to regulate dysphoric moods or to escape the frustrations of daily life.
Loss aversion	7-35	Tendency of a loss to frustrate or to “spoil the fun.” Likely subsumes a search for a challenge.
Customization	4-20	Interest in in-game creative pursuits such as personalizing an in-game avatar or building a house.
Grinding	6-30	Attitudes toward performing repetitive actions or paying real-life money to earn in-game rewards; interest in performing every possible action in a game or collecting every in-game item.
Autonomy	5-25	Enthusiasm for games with many choices, options, multiple solutions to puzzles, and open areas to explore.

^a^GAMES: Gaming Attitudes, Motivations, and Experiences Scales.

#### Translations of the Instruments and a Pilot Study

The original version of GAMES was written in English; thus, a translation and back translation were performed to ensure that the validity was preserved [41]. First, the original instrument was translated into traditional Chinese by the researchers. Then, a language expert translated it back into English. Third, the original and the translated English versions were compared to ensure that the validity was retained after the translation [41]. In cases of any discrepancy between the original and the translated versions, the translation was reassessed by a researcher, translator, and the original author of GAMES.

A pilot study was conducted to examine the data collection procedure and reliability of the instrument [53]. Meanwhile, the feasibility of the data collection was tested [54]. We recruited 20 people with schizophrenia who were living in their community in Hong Kong. Two identical 2-way translated questionnaires were distributed to participants with an interval of 2 weeks [55]. The intraclass correlation coefficient was assessed to identify the test-retest reliability, and the Cronbach α was assessed to identify the internal consistency of the instrument [53,54]. The test-retest reliability was moderate to good with an intraclass correlation coefficient of 0.58-0.95. The internal consistency was good with a Cronbach α of .71-.94. The reliability of the translated version was comparative to that of the original GAMES.

### Data Collection

The data collection period lasted for 16 weeks—from October 2017 to January 2018. A researcher promoted the study with posters and arranged venues for briefing and data collection. Identical sessions were run by the same researcher at different time points at each data collection site [41]. All eligible participants were invited to attend an information session organized together with the nongovernmental organization. Potential participants were informed about the purpose of the study, the rights of the participants, risks of participation, and possible benefits, both orally and in written format. They were also informed about their right to refuse and withdraw from the study at any time without any consequences [45]. After informed consent forms were signed, the questionnaire was distributed to each participant. After completion, questionnaires were collected using sealed envelopes [41]. Each participant received a supermarket redemption coupon worth HKD $50 (US $6.45) as compensation for their participation in the study. Data collection sessions lasted for 30-60 minutes [32]. A total of 18 data collection sessions were held at the 13 data collection sites. Finally, 148 people were contacted and invited to participate in the study, and 110 (74.3%) eligible people were willing to participate in the study and they completed the questionnaire.

### Data Analysis

First, collected data were processed through data input, data cleansing, and data reversing [41]. Second, frequencies, percentages, maximum score, minimum score, mode, median, mean, and standard deviation were used to describe the demographic characteristics and the participants’ habits and attitudes regarding internet use and video gaming [52]. To facilitate the analysis of the current and favorite video games played, the entries were recategorized into different game genres. We utilized an extensive gaming archive [56] to sort the entries into 10 mutually exclusive game genres. Lastly, a two-tailed independent *t* test was used to describe the possible differences in the normally distributed variables of the gaming attitudes. The Pearson’s correlation coefficient was used to quantify the linear relationship between the categories of the gaming attitudes. Statistical significance was established at *P*=.05. For the dimensions of the categories of the gaming attitudes that were the most intercorrelated, principal analysis with 3 methods of rotation (varimax, equamax, and promax) was used to confirm the convergence of the 9 categories of gaming attitudes into fewer principle factors for easy interpretation. The SPSS software for Windows 22.0 (IBM Corp) was used for the data analysis.

## Results

### Background Information of the Participants

The mean age of the 110 participants was 39.0 (SD 11.2) years. About two-thirds of the participants (70/110, 63.6%) were males. A clear majority (91/110, 82.7%) was single and had completed high school or higher education (73/110, 66.4%). Totally 26.4% (29/110) of the participants were receiving social welfare benefits, 18.2% (20/110) were receiving disability pension, while 25.4% (28/110) were employed and 23.6% (26/110) were unemployed. Of the 110 participants, 78 (70.9%) lived in supported housing (Table 2). The mean reported age at the first contact with psychiatric services was 24.7 (SD 8.5) years, and the average duration of illness was 14.3 (SD 10.3) years.

### Internet Use

The majority of the participants had access to an internet connection (100/110, 90.9%). The most common purposes of using the internet were to watch videos (66/110, 60.0%), read the news, papers, magazines, and books on the internet (42/110, 38.2%), research information about other topics or issues of interest to them (41/110, 37.3%), and play web-based games (36/110, 32.7%). Participants found using the internet to be easy (69/110, 62.7%). The major causes that lessened the use of the internet were when eyes were tired easily (40/110, 36.4%) and when one had difficulty concentrating for long periods of time (26/110, 23.6%) (Table 3).

**Table 2:** Background information of the participants (N=110).

Variables		Values, n (%)
**Gender**		
	Male	70 (63.6)
	Female	40 (36.4)
**Marital status**		
	Single	91 (82.7)
	Partnership/Married	9 (8.2)
	Separated/Divorced	10 (9.1)
**Level of education**		
	Primary and middle school	37 (33.6)
	High school	51 (46.4)
	College or vocational training	10 (9.1)
	Bachelor’s degree	12 (10.9)
**Employment status**		
	Unemployed	26 (23.6)
	Social welfare benefit	29 (26.4)
	Student	2 (1.8)
	Disability pension	20 (18.2)
	Employed (including sick leave)	28 (25.5)
	Other	5 (4.5)
**Living situation**		
	Own household (with partner/family)	8 (7.3)
	Flat share	17 (15.4)
	Parents’ household	7 (6.4)
	Supported housing	78 (70.9)

**Table 3:** Patterns of internet use among the participants (N=110).

Patterns of internet use		Values, n (%)
**Access location of the internet**		
	Home	77 (70.0)
	Apartment complex	5 (4.5)
	Family/friend’s home	9 (8.2)
	Library	4 (3.6)
	Other	5 (4.5)
	Not using the internet at all	10 (9.1)
**Possession of an email address**		
	Yes	63 (57.3)
	No	47 (42.7)
**Frequency of using internet**		
	At least once a day	54 (49.1)
	Every few days	14 (12.7)
	Once a week	6 (5.5)
	A few times a month	3 (2.7)
	Once a month or less	33 (30.0)
**Purpose of internet use**		
	Research health-related information	18 (16.4)
	Communicate with health professionals about health-related issues	4 (3.6)
	Communicate with other users about health-related issues	8 (7.3)
	Research information about other topics or issues of interest to me	41 (37.3)
	Send/receive email	24 (21.8)
	Online shopping	15 (13.6)
	Perform online banking/pay bills	13 (11.8)
	Read web-based news, papers, magazines, and books	42 (38.2)
	Play web-based games	36 (32.7)
	Watch videos	66 (60.0)
	Use social networking websites and/or dating sites	24 (21.8)
	Other	9 (8.2)
**Easiness to access the intended information on the internet**		
	Always easy	22 (20.0)
	Sometimes easy	47 (42.7)
	Not so easy	17 (15.5)
	Difficult	11 (10.0)
	Very difficult	13 (11.8)
**Problems that lessen internet use**		
	Pain in the limbs	7 (6.4)
	Unsteady hands	6 (5.5)
	Difficulty concentrating for long periods of time	26 (23.6)
	Difficulty sitting for long periods of time	18 (16.4)
	Eyes that tire easily	40 (36.4)
	Other problems	6 (5.5)

### Habits of Video Gaming

The majority of the participants (88/110, 80.0%) reported that they had played a video game within the past year. The habits of video gaming of the 88 participants were explored (Table 4). Almost half of the participants (41/88, 47%) regarded themselves as playing games seriously or very seriously. Half of our participants played video games on a daily (22/88, 25%) to weekly basis (26/88, 30%). The most favorable gaming platforms were cellular phones (43/88, 49%), computers (19/88, 22%), and arcade cabinets (6/88, 7%) (Table 4). Referring to the video games that participants were currently playing and their favorite video games, there were 118 and 145 entries, which included 81 and 84 games, respectively (Table 5). The favorite game genre among the participants was that of action games.

**Table 4:** Habits of video gaming of the participants (n=88).

Habits of video gaming			Values, n (%)
**Seriousness of video gaming**			
	Very serious	14 (16)	
	Serious	27 (31)	
	Neither serious nor casual	17 (19)	
	Casual	25 (28)	
	Very casual	5 (6)	
**Frequency of video gaming**			
	Daily	22 (25)	
	2-3 times per week	15 (17)	
	Weekly	11 (13)	
	2-3 times per month	11 (13)	
	Monthly	8 (9)	
	Less than monthly	21 (24)	
**Proportion of spare time spent on video gaming**			
	Almost all of the spare time	4 (5)	
	Most of the spare time	6 (7)	
	Some of the spare time	27 (31)	
	Less of the spare time	30 (34)	
	Almost none of the spare time	21 (24)	
**The most typical media platform used to play games**			
	Cellular phone	43 (49)	
	Computer	19 (22)	
	Arcade cabinets	6 (7)	
	Facebook	3 (3)	
	Board or card games (tabletop games)	3 (3)	
	Sony PlayStation	2 (2)	
	Sony PlayStation Portable	2 (2)	
	Microsoft Xbox	2 (2)	
	Real-life sports	2 (2)	
	Nintendo Wii	1 (1)	
	Nintendo DS	1 (1)	
	Not specified	4 (5)	

**Table 5:** Video games played by the participants.

Game genres	Currently played game genres (n=118), n (%)	Favorite game genres (n=145), n (%)
Action	23 (19.5)	34 (23.4)
Strategy	23 (19.5)	26 (17.9)
Miscellaneous (board/card games)	20 (17.0)	27 (18.6)
Role-playing	15 (12.7)	17 (11.7)
Simulation	14 (11.9)	13 (9.0)
Puzzle	10 (8.5)	11 (7.6)
Sports	9 (7.6)	13 (9.0)
Action adventure	3 (2.5)	3 (2.1)
Adventure	1 (0.8)	N/A^a^
Rhythm	N/A	1 (0.7)

^a^Not applicable.

### Gaming Attitudes

The gaming attitudes of the participants were explored, and the results are summarized in Table 6. Of the 88 participants, the distribution of the scores in the category of story ranged from 21 to 50 with a mean score of 37.81 (SD 6.33) (Table 6).

To compare the scores among the 9 attitude measures, we standardized the raw score of each participant as a relative score for each attitude measure. The mean of the standard score in the last column of Table 6 suggests that the top 3 favorable video gaming categories for participants were autonomy, escapism, and story, and the last two were violent catharsis and violent reward. Furthermore, the gaming attitudes of the participants were mostly correlated with each other (Table 7). A principal analysis with 3 methods of rotation (varimax, equamax, and promax) indicated that the categories of story, social interaction, customization, and autonomy formed one factor, which are related to social playing and explains 13.1% of the variation, and the rest of the categories formed another factor, which are related to evasive playing, thereby explaining 45.7% of the variation.

**Table 6:** A summary of the gaming attitudes of the participants (n=88).

Categories		Minimum score		Median score		Mode		Maximum score		Mean (SD)		Mean of standard score in %^a^ (rank)	
**Social playing**													
	Story		21		38		37		50		37.81 (6.33)		54 (3)
	Social interaction		6		19		20		30		18.55 (4.78)		52 (5)
	Customization		4		12		12		20		12.05 (3.89)		50 (6)
	Autonomy		6		17		15		25		16.67 (4.01)		58 (1)
**Evasive playing**													
	Violent catharsis		7		21		14, 21		35		19.92 (5.91)		46 (8)
	Violent reward		6		16		18		30		15.19 (5.44)		38 (9)
	Escapism		6		20		18		30		19.16 (4.92)		55 (2)
	Loss aversion		8		21		22		33		20.74 (4.60)		49 (7)
	Grinding		8		18		18		30		18.88 (4.09)		54 (4)

^a^Standard score is defined as Absolute (actual score – minimum value)/theoretical range.

**Table 7:** Pearson’s correlation coefficients and *P* values among the video gaming attitude measures (n=88).

Variable		Violent catharsis	Violent reward	Social interaction	Escapism	Loss aversion	Customization	Grinding	Autonomy
**Story**									
	*r*	0.23^a^	0.22^a^	0.37^b^	0.28^b^	–0.05	0.33^b^	0.30^b^	0.38^b^
	*P* value	.03	.04	<.001	.008	.67	.002	.004	<.001
**Violent catharsis**									
	*r*	—^c^	0.44^b^	0.32^b^	0.40^b^	0.46^b^	0.40^b^	0.53^b^	0.39^b^
	*P* value	—	<.001	.003	<.001	<.001	<.001	<.001	<.001
**Violent reward**									
	*r*	—	—	0.20	0.26^a^	0.24^a^	0.21^a^	0.31^b^	0.17
	*P* value	—	—	.06	.02	.02	.05	.004	.11
**Social interaction**									
	*r*	—	—	—	0.40^b^	0.20	0.54^b^	0.52^b^	0.38^b^
	*P* value	—	—	—	<.001	.06	<.001	<.001	<.001
**Escapism**									
	*r*	—	—	—	—	0.53^b^	0.44^b^	0.52^b^	0.59^b^
	*P* value	—	—	—	—	<.001	<.001	<.001	<.001
**Loss aversion**									
	*r*	—	—	—	—	—	0.30^b^	0.48^b^	0.38^b^
	*P* value	—	—	—	—	—	.005	<.001	<.001
**Customization**									
	*r*	—	—	—	—	—	—	0.60^b^	0.50^b^
	*P* value	—	—	—	—	—	—	<.001	<.001
**Grinding**									
	*r*	—	—	—	—	—	—	—	.74^b^
	*P* value	—	—	—	—	—	—	—	<.001

^a^The correlation is significant at a significance level of .05 (two-tailed).

^b^The correlation is significant at a significance level of .01 (two-tailed).

^c^Not applicable.

### Association of the Background Information with Gaming Attitudes

Several background characteristics such as marital status, education level, and employment status were found to be significantly associated with several categories of gaming attitudes (Multimedia Appendix 1). As for marital status, participants who were married or who were in a partnership had a higher score than those who were single, separated, or divorced in the violent catharsis category (t_86_=2.66, *P*=.01). With regard to the education level, participants who held a bachelor’s degree or higher had a lower score than those who were educated with college or vocational training or a lower level of education in the violent reward category (t_86_=–2.57, *P*=.01). Likewise, the participants with a bachelor’s degree or higher had a lower score in social interaction than those who did not (t_86_=–2.25, *P*=.03). Further, the participants who had a bachelor’s degree had a lower score in customization than those who did not (t_86_=–2.08, *P*=.04). Regarding the employment status, participants who were employed or studying scored lower than those who were unemployed or others in the violent reward category (t_86_=–3.11, *P*=.003). Similarly, participants who were employed or studying scored lower than those who were unemployed or others in the loss aversion category (t_86_=1.98, *P*=.05).

The gaming habits of the participants were also found to have a significant association with several gaming attitudes (Multimedia Appendix 1). Regarding the seriousness of video gaming, noncasual players had a higher score than casual players in the customization category (t_86_=2.00, *P*=.05). Participants who played video games on a daily basis scored higher on story than those who played less than daily (t_86_=2.03, *P*=.05). Participants who reported that they spent more spare time on video gaming (almost all/most of/some of their spare time) scored higher on violent reward than those who spent less spare time (few/almost none of their spare time) (t_86_=2.14, *P*=.04). Those who spent more spare time on video gaming also scored higher in escapism than those who spent less spare time on gaming (t_86_=2.79, *P=*.007). However, no significant correlation was found between age, the age at the first contact with psychiatric services, or duration of illness and any of the categories of the gaming attitudes.

The analysis (Multimedia Appendix 2) based on the factor loading weighted mean score of the 2 summarized factors of the gaming attitudes, “social playing” and “evasive playing” provided further support for the above findings in a summarized manner. This analysis suggested that participants with a bachelor’s degree or above and employed or studying seemed to have lower scores in social playing than their counterparts, thereby reflecting a difference in their attitude regarding social interaction. However, the differences were not statistically significant owing to the sample being small. Participants employed or studying had significantly low scores in evasive playing (t_86_=2.16, *P=*.03), which is a reflection of the significant differences in their attitudes on violent reward and loss aversion. The notable difference between the 2 gaming attitude components was that those in partnership/marriage scored much higher in evasive playing than single/separated/divorced individuals (68.73 vs 61.94, respectively), although they scored similarly in social playing (60.62 vs 61.38, respectively), that is, the difference was not significant in this study. The differences in the levels of seriousness and frequency were similar to the differences between the 2 factors of attitudes, but there was a larger difference in the proportion of spare time spent on video gaming; more spare time was spent on evasive playing than on social playing, with a borderline significance of t_86_=1.94, *P=*.06.

## Discussion

### Principal Findings

This study is the first to describe the habits and attitudes of internet use and video gaming among people with schizophrenia in an Asian city. Similar to the global trends, the majority of the participants (100/110, 90.9%) utilized the internet with adequate skills and participated in video gaming [6]. Their favorite gaming platform and game genres were cellular phones and action games, respectively. However, only few of the participants had utilized the internet for seeking health information, and the efficacy of health education through web-based platforms to this population was limited. The gaming attitudes were identified and found to be associated with several characteristics. The participants valued autonomy in gaming and disfavored violence in video games. Through our analysis, we established the motives of the gamers. “Social playing” included social interaction, story, customization, and autonomy, while “evasive playing” included grinding, loss aversion, escapism, violent catharsis, and violent reward.

### Use of Information Technology

The utilization of information technology was found to extend to almost the entire population of Hong Kong, which is a modern city [57]. We found that the frequency of internet use by most of our participants was higher than those reported in the United States [29] and Finland [30]. The high accessibility to the internet could be explained by the affordable and extensive coverage of the internet network [58]. As reported in previous studies [6,30], our participants mainly utilized the internet to watch videos and obtain information by reading news, papers, magazines, and books. Over half of the participants (69/110, 62.7%) found it easy to obtain the intended information from the internet. Our study also demonstrates that most of the participants were able and willing to obtain information from the internet.

Despite the availability of web-based resources and the ability to use them, only 16.4% (18/110) of the study participants reported utilizing the internet for researching health-related information. This may imply that the efficacy of mental health promotion through web-based platforms is still limited. McCloud et al [59] suggest that the low motivation of internet users to search for health-related information might be due to users lacking the skills needed to gather and identify trustworthy sources of information. Athanasopoulou et al [60] and Schrank et al [33] also demonstrated that the quality of most of the easily accessed information sources about schizophrenia on the internet is poor. This can hinder internet users from receiving useful and nonstigmatized information about managing schizophrenia. It would be preferable for health care authorities to develop and use a trustworthy internet platform through which health-related information for health promotion could be easily distributed. Furthermore, our results reflect that there are physical constraints that pose barriers to the use of the internet. The major barriers were found to be tired eyes and concentration difficulties. The adverse effects of antipsychotic drugs, such as drowsiness, sedation, and anticholinergic effects, might be a cause of the barriers [61]. Thus, it is important for health care staff to address safe methods in promoting health-related issues through trusted web-based platforms on a daily basis.

### Habits of Video Gaming

Video gaming is popular in Hong Kong, and approximately 94% of the adolescents comprise the gaming population [62]. Likewise, a majority of the participants had played video games within the past year (88/110, 80%). As reported globally [63], the most popular gaming platform among the participants was cellular phones. There has been a global shift toward playing games on cellular phones rather than sitting in front of a television or a desktop computer. With the high mobility and convenience of using phones, gamers can enjoy playing without geographical constrictions. Indeed, game developers have launched numerous games on mobile platforms in recent years [6,63]. However, since players use a limited-sized screen and gaming times are generally short in mobile gaming, designing games for mobile phones with long and complex stories is not preferred [62].

The participants also played video games frequently; more than half of them (48/88, 55%) played at least once a week and (22/88, 25%) played on a daily basis. About half of the gamers emphasized on video gaming, as they played seriously. As reflected in the best-selling video games of 2017, action games were the favorite game genre among the participants. Action games challenge gamers through physical means and players usually control a character to complete tasks that require a certain level of hand-eye coordination and skills [6,64]. Action games attract players by rewarding them with excitement [62]. Contrary to the global best-selling trends, our results indicated that card and board games and strategy games were the next favorite types of games, while the best-selling games worldwide were role-playing and sports games [6]. This finding might be accounted for by the cultural and local context. Local game developers in Hong Kong have converted some culturally traditional board games such as Mahjong, Chinese Chess, and some card games into digital versions, which are easy to use. In addition, some traditional Chinese stories such as “Romance of the three kingdoms” has been converted to a series of strategy games. However, the characteristics of the players in Hong Kong do not seem to differ significantly from those of the rest of the world with regard to popular mobile games [63]. Thus, one possible explanation for our finding of people with schizophrenia favoring traditional games could be related to the negative symptoms of the disease, such as cognitive deficits and a lack of confidence about one’s own abilities [64]. These factors could lead to favoring familiar game genres.

### Gaming Attitudes

Our study identified that autonomy was the most common motive for video gaming among the participants. This is seen in the preference of the participants to play games in which they could manipulate settings and make choices. Video games provide a normalized platform wherein people can exercise self-sufficiency and thus develop self-competence [65]. The next most common motive was escapism, which implies that participants played games to regulate dysphoria and escape from the hassles of daily life. Indeed, video gaming has been considered a culturally acceptable way to relieve the stress of reality [23,25]. On the other hand, some scholars argue that escapism is associated with gaming addiction and adverse emotional experiences [66-69]. A recent study examined the effects of video gaming on a group of military veterans with mental problems and found that video gaming contributed to their recovery by promoting individuals’ adaptive coping, eudaimonic well-being, and socializing [70]. As the evidence related to harm caused by gaming among people with schizophrenia is not yet conclusive, staff working in clinical practice need to be sensitive to and be aware of any potential harmful effects of the interventions and avoid any such threats.

Our participants regarded violent reward and violent catharsis as the least favorable motives for video gaming. Numerous studies suggest that violent games might contribute to aggression [70-72] and moral desensitization [73,74]. On the contrary, scholars also suggest that violent games support emotional coping after stressful events [75,76]. Interestingly, our results indicate that people with schizophrenia do not favor violence in video gaming. Moreover, several characteristics were associated with a preference of violent video gaming. People who were married or in a partnership tended to channelize their negative emotions by playing violent games to avoid hurting others. In contrast, the unemployed and those with lower education levels enjoyed the satisfaction they received from playing violent games. These results implied that people with different sociodemographic characteristics achieve emotional coping through different ways. However, more studies are needed with larger sample sizes to confirm valid links between sociodemographic information and gaming motives.

Studies have shown that persons with schizophrenia are lonely and isolated and less motivated to socially engage with others [3-7]. Our analysis regarding gamers’ motives identified a factor, “social playing,” in several categories, namely, social interaction, story, customization, and autonomy. This factor refers to people who emphasize social processes in video gaming. These players enjoy games that enable them to customize and manipulate numerous settings. The story and settings of the video games strongly influence the gaming experience for the players. Gamers value the social interaction with friends and other players during the gaming process. Likewise, several background characteristics were related to social playing motives. In the past decade, there has been an expansion of multiplayer web-based role-playing games in the market. These have provided a virtual platform for players to interact with people worldwide [6,55,62,77]. Scholars have proven that the formation of social bonds in virtual communities through web-based video games facilitates the development of interpersonal skills in the real world [78,79]. This shows the potential of utilizing video games, regardless of the players’ education levels, to facilitate interpersonal skills among people with mental illness [80,81] and to increase feelings of relatedness [36]. Frequent game players in our data were more concerned with the game story. Likewise, noncasual players and people with a lower education level preferred games with customizable settings. With the rapid expansion of the gaming market, gamers have a higher expectation of the quality of games, including that of the story, settings, ability to personalize, and the gaming experience [82]. Indeed, gamers can exercise creativity and control their game by tailor-making their own game settings. Studies have shown that the ability to personalize games motivates people to play games [82-84].

On the contrary, another factor, “evasive playing,” was formed by the remaining categories, namely, grinding, loss aversion, escapism, violent catharsis, and violent reward. Evasive playing refers to when gamers emphasize the rewards from video gaming and spend time on achieving all gaming missions and collecting all possible props in a game. In this style of playing, the violence in games is utilized to channel negative emotions and attain enjoyment. Our results indicate that unemployed people have a high loss-aversion score, which could imply that gaming is significantly important to them. People who spent more spare time on gaming were more likely to utilize gaming to escape from the hassles of daily life. However, scholars suggest that these motives are linked to problematic use [15,85] and pathological gaming [32], which should be seriously considered when discussing gaming among people with mental illness. In the future, more studies should therefore be conducted in schizophrenic patient populations with increased sample sizes and a longitudinal design and follow-up to explore problematic uses of video games, both in clinical and community settings.

### Strengths and Limitations

This study fills a knowledge gap by depicting a pattern of internet use and video gaming among people with schizophrenia. Despite the research aims being met, there are some limitations to address. First, participant recruitment was conducted using a convenience sample. Thus, the sample size was small, which limits the generalization of the results to the wide population of video game players in Hong Kong or in other parts of the world. Second, our participants were recruited from 2 nongovernmental organizations (supported housing); therefore, the results may be biased toward individuals who live in communities with better general levels of mental health. Third, the study may have attracted people who were interested and skillful in video gaming. Further, the life span and the popularity of video games are highly influenced by the social trends that might change rapidly.

### Implications

The utilization of the internet as a platform for health promotion and education has been considered to be a cost-effective measure [86,87]. Most people with schizophrenia in our study were found to be able to utilize information technology to obtain information on the internet. This finding is promising. Based on a systematic review by Suenderhauf et al [15], regular video game playing could enable home monitoring of patients. For example, staff could use gaming as a tool for patient monitoring in order to initiate supportive measures to avoid relapses. Decreased login times could indicate diminishing compliance or worsening of disease. Likewise, alarming habits in computer use such as the lack of logins in the regular gaming schedule or playing beyond suggested gaming hours could be an indicator for health care professionals of changes in the patients’ mental status. However, this type of monitoring would require specified technological devices and access for patients who play games remotely, which may increase potential concerns in patient privacy and security issues. It is therefore important to delineate the roles and responsibilities of health professionals in patient monitoring in any exceptional situation (eg, suicide attempt). Professionals should also support the use of digital health interventions; their attitudes are crucial for successful implementation of health technology in patient daily care [88]. Our previous studies have shown that staff members may still be less willing to adopt new technology-based interventions into mental health care [89,90] as they tend to prefer traditional face-to-face methods in mental health care services [91].

We found that the internet was less often used to seek health-related information. Scholars and health care professionals might need to investigate the cause of the low utilization rate of the internet with regard to searching for health-related information. To promote internet use in a population, health care authorities should consider developing an internet platform with high-quality health information for health promotion. Service organizations should provide equipment, training, and ongoing information technology support to the population in order to expand their social networks and maximize self-help abilities. Policy makers should promote the use of information technology in a schizophrenic population and offer support to the nongovernmental organizations for the abovementioned measures.

Over the last decade, numerous studies have shown video gaming as an intervention for various medical conditions. From the mental health aspect, health care professionals could utilize video games to promote patient engagement, stress coping, and social skills training. Our findings show that game developers could design video games to fit the needs of the population. Game developers and health care professionals could refer to the identified gaming attitudes to tailor-make attractive serious games for populations with mental health problems accordingly. Therefore, in the future, more studies should be conducted with more diverse samples to identify the potential benefits of video gaming for patients with mental problems. The use of health-related games should also be implemented and tested as part of the mental health recovery. Despite the huge potential that information technology has in the treatment of patients with mental disorders, we still may have a long way to go before research on computer-based interventions can realize its full potential in psychiatric services [92].

### Conclusion

This study demonstrates that the internet utilization rate of people with schizophrenia was as high as that of the general population in Hong Kong. Relatively few of them used the internet to search for health-related information. Further investigation into the underlying reasons would be vital to mental health promotion through web-based platforms. Our study also exemplifies the unique habits of gaming among the study participants. Health care professionals could utilize video games to engage patients, promote stress coping, and provide social skills training. The identified gaming attitudes can be used to contribute to the development of serious games for the population in the future.

## Appendix

Multimedia Appendix 1 Associations between background characteristics and video gaming attitudes.

Multimedia Appendix 2 Associations between background characteristics and principle components of video gaming attitudes.

## Abbreviations

GAMES: Gaming Attitudes, Motivations, and Experiences Scales
