# Supplementary material for: Habits and Attitudes of Video Gaming and Information Technology Use in People with Schizophrenia: Cross-Sectional Survey
Source: J Med Internet Res. 2020 Jul 22;22(7):e14865. doi: 10.2196/14865 (PMC7407262; doi:10.2196/14865)
Supplement: Multimedia Appendix 1 [file jmir_v22i7e14865_app1.docx]

Multimedia appendix 1. Associations between background characteristics and video gaming attitudes

|  | **Story** | | **Violent catharsis** | | **Violent reward** | | **Social interaction** | | **Escapism** | | **Loss-aversion** | | **Customization** | | **Grinding** | | **Autonomy** | |
| --- | --- | --- | --- | --- | --- | --- | --- | --- | --- | --- | --- | --- | --- | --- | --- | --- | --- | --- |
|  | Mean (SD) | *P* | Mean (SD) | *P* | Mean (SD) | *P* | Mean (SD) | *P* | Mean (SD) | *P* | Mean (SD) | *P* | Mean (SD) | *P* | Mean (SD) | *P* | Mean (SD) | *P* |
|  |  |  |  |  |  |  |  |  |  |  |  |  |  |  |  |  |  |  |
| **Gender** |  | .59 |  | .75 |  | .22 |  | .44 |  | .68 |  | .99 |  | .88 |  | .69 |  | .22 |
| Male | 38.07 (6.73) |  | 20.07 (5.88) |  | 15.71 (5.38) |  | 18.26 (4.62) |  | 19.00 (4.91) |  | 20.74 (4.46) |  | 12.00 (3.84) |  | 19.00 (4.21) |  | 16.95 (3.70) |  |
| Female | 37.30 (5.53 |  | 19.63 (6.07) |  | 14.20 (5.52) |  | 19.10 (5.10) |  | 19.47 (5.00) |  | 20.73 (4.94) |  | 12.13 (4.05) |  | 18.63 (3.93) |  | 15.83 (4.53) |  |
| **Marital Status** |  | .40 |  | .009^b^ |  | .79 |  | .50 |  | .21 |  | .45 |  | .64 |  | .92 |  | .63 |
| Single/ Separated/ Divorced | 37.98 (6.13) |  | 19.44 (5.77) |  | 15.15 (5.45) |  | 18.44 (4.79) |  | 18.96 (4.93) |  | 20.63 (4.55) |  | 11.99 (3.88) |  | 18.89 (4.01) |  | 16.63 (3.90) |  |
| Partnership/ Married | 35.86 (8.59) |  | 25.43 (4.93) |  | 15.71 (5.79) |  | 19.71 (4.86) |  | 21.43 (4.43) |  | 22.00 (5.45) |  | 12.71 (4.27) |  | 18.71 (5.38) |  | 15.86 (5.49) |  |
| **Education level** |  | .58 |  | .11 |  | .010^b^ |  | .03^a^ |  | .92 |  | .93 |  | .04^a^ |  | .18 |  | .75 |
| College/ Vocational training or below | 37.93 (6.29) |  | 20.24 (5.81) |  | 15.65 (5.37) |  | 18.90 (4.64) |  | 19.18 (4.93) |  | 20.73 (4.70) |  | 12.31 (3.77) |  | 19.06 (4.02) |  | 16.61 (4.12) |  |
| Bachelor degree or above | 36.63 (6.97) |  | 16.75 (6.43) |  | 10.63 (4.07) |  | 15.00 (4.96) |  | 19.00 (5.07) |  | 20.88 (3.76) |  | 9.38 (4.34) |  | 17.00 (4.63) |  | 16.13 (2.90) |  |
| **Employment status** |  | .15 |  | .35 |  | .003^b^ |  | .13 |  | .11 |  | .05^a^ |  | .15 |  | .14 |  | .81 |
| Employed/ Student | 36.17 (7.11) |  | 18.91 (6.27) |  | 12.30 (4.73) |  | 17.26 (5.61) |  | 17.74 (6.35) |  | 19.13 (4.98) |  | 11.04 (4.55) |  | 17.78 (5.02) |  | 16.39 (4.54) |  |
| Unemployed/ Other | 38.38 (5.98) |  | 20.28 (5.79) |  | 16.22 (5.34) |  | 19.00 (4.41) |  | 19.66 (4.25) |  | 21.31 (4.36) |  | 12.40 (3.60) |  | 19.26 (3.68) |  | 16.63 (3.84) |  |
| **Living situation** |  | .92 |  | .16 |  | .26 |  | .46 |  | .47 |  | .71 |  | .32 |  | .24 |  | .59 |
| Household (with partner/ family)/ Flat share | 37.70 (5.80) |  | 18.43 (5.74) |  | 14.09 (4.86) |  | 17.91 (4.61) |  | 18.52 (4.97) |  | 21.04 (4.70) |  | 11.35 (3.69) |  | 18.00 (3.53) |  | 16.17 (3.56) |  |
| Supported housing | 37.85 (6.55) |  | 20.45 (5.93) |  | 15.58 (5.62) |  | 18.77 (4.85) |  | 19.38 (4.92) |  | 20.63 (4.60) |  | 12.29 (3.96) |  | 19.18 (4.26) |  | 16.71 (4.17) |  |
| **Internet connection at home** |  | .84 |  | .78 |  | .15 |  | .40 |  | .66 |  | .83 |  | .81 |  | .91 |  | .89 |
| Yes | 37.89 (6.22) |  | 19.81 (5.50) |  | 15.70 (5.46) |  | 18.28 (4.50) |  | 19.02 (4.62) |  | 20.67 (4.20) |  | 11.98 (3.86) |  | 18.91 (4.10) |  | 16.53 (3.72) |  |
| No | 37.58 (6.73) |  | 20.21 (7.03) |  | 13.93 (5.26) |  | 19.25 (5.49) |  | 19.54 (5.73) |  | 20.92 (5.64) |  | 12.21 (4.03) |  | 18.79 (4.15) |  | 16.67 (4.79) |  |
| **Email address** |  | .69 |  | .72 |  | .11 |  | .23 |  | .57 |  | .79 |  | .77 |  | .58 |  | .63 |
| Yes | 38.04 (5.91) |  | 19.73 (5.30) |  | 15.98 (5.31) |  | 18.02 (4.45) |  | 18.90 (5.01) |  | 20.63 (4.09) |  | 11.94 (3.93) |  | 18.67 (4.18) |  | 16.39 (3.70) |  |
| No | 37.49 (6.93) |  | 20.19 (6.74) |  | 14.11 (5.51) |  | 19.27 (5.17) |  | 19.51 (4.84) |  | 20.89 (5.28) |  | 12.19 (3.88) |  | 19.16 (4.00) |  | 16.81 (4.44) |  |
| **Seriousness on video gaming** |  | .95 |  | .15 |  | .69 |  | .47 |  | .56 |  | .80 |  | .05^a^ |  | .15 |  | .78 |
| Non-casual player | 37.78 (6.79) |  | 20.57 (5.74) |  | 15.36 (5.51) |  | 18.81 (4.79) |  | 19.38 (4.34) |  | 20.83 (4.49) |  | 12.64 (3.83) |  | 19.33 (4.26) |  | 16.48 (3.92) |  |
| Casual player (Casual/ Very casual) | 37.87 (5.44) |  | 18.67 (6.14) |  | 14.87 (5.39) |  | 18.03 (4.79) |  | 18.73 (4.93) |  | 20.57 (4.89) |  | 10.90 (3.81) |  | 18.00 (3.67) |  | 16.73 (4.25) |  |
| **Frequency on video gaming** |  | .05^a^ |  | .66 |  | .17 |  | .96 |  | .17 |  | .61 |  | .17 |  | .24 |  | .34 |
| Daily | 40.14 (5.51) |  | 20.41 (5.40) |  | 16.59 (5.20) |  | 18.59 (5.59) |  | 20.41 (4.79) |  | 21.18 (5.03) |  | 13.05 (4.27) |  | 19.77 (4.48) |  | 17.27 (4.61) |  |
| Less than daily | 37.03 (6.43) |  | 19.76 (6.10) |  | 14.73 (5.48) |  | 18.53 (4.52) |  | 18.74 (4.93) |  | 20.59 (4.48) |  | 11.71 (3.73) |  | 18.58 (3.95) |  | 16.33 (3.80) |  |
| **Proportion of spare time spent on video gaming** |  | .50 |  | .41 |  | .04^a^ |  | .73 |  | .007^b^ |  | .29 |  | .22 |  | .28 |  | .71 |
| Use more of one's spare time | 38.35 (6.53) |  | 20.54 (5.07) |  | 16.62 (6.14) |  | 18.76 (5.20) |  | 20.81 (4.37) |  | 21.35 (5.26) |  | 12.65 (3.77) |  | 19.43 (4.51) |  | 16.76 (4.27) |  |
| Use less of one's spare time | 37.41 (6.21) |  | 19.47 (5.81) |  | 14.16 (4.67) |  | 18.39 (4.49) |  | 17.96 (4.98) |  | 20.29 (4.06) |  | 11.61 (3.95) |  | 18.47 (3.76) |  | 16.43 (3.85) |  |

*^a^ P≤.05*

*^b^P≤.01*
