# Supplementary material for: Habits and Attitudes of Video Gaming and Information Technology Use in People with Schizophrenia: Cross-Sectional Survey
Source: J Med Internet Res. 2020 Jul 22;22(7):e14865. doi: 10.2196/14865 (PMC7407262; doi:10.2196/14865)
Supplement: Multimedia Appendix 2 [file jmir_v22i7e14865_app2.docx]

Appendix 2 Associations between background characteristics and principle components of video gaming attitudes

|  |  | **Social Playing** | |  | **Evasive Playing** | |
| --- | --- | --- | --- | --- | --- | --- |
|  | N | Weighted mean (SD) | *P* |  | Weighted mean (SD) | *P* |
| **Gender** |  |  | .80 |  |  | .59 |
| Male | 58 | 61.52(10.25) |  |  | 62.98(11.63) |  |
| Female | 30 | 60.93(9.95) |  |  | 61.51(12.79) |  |
| **Marital Status** |  |  | .85 |  |  | .15 |
| Single/ Separated/ Divorced | 81 | 61.38(10.16) |  |  | 61.94(11.93) |  |
| Partnership/ Married | 7 | 60.62(10.10) |  |  | 68.73(11.71) |  |
| **Education level** |  |  | .11 |  |  | .24 |
| College/ Vocational training or below | 80 | 61.87(9.84) |  |  | 62.95(12.03) |  |
| Bachelor degree or above | 8 | 55.80(11.66) |  |  | 57.73(11.09) |  |
| **Employment status** |  |  | .10 |  |  | .03* |
| Employed/ Student | 23 | 58.32(9.75) |  |  | 57.93(13.71) |  |
| Unemployed/ Other | 65 | 62.37(10.08) |  |  | 64.09(10.98) |  |
| **Living situation** |  |  | .50 |  |  | .36 |
| Household (with partner/ family)/ Flat share | 23 | 60.08(8.79) |  |  | 60.49(10.47) |  |
| Supported housing | 65 | 61.75(10.57) |  |  | 63.18(12.47) |  |
| **Internet connection at home** |  |  | .78 |  |  | .99 |
| Yes | 64 | 61.13(9.97) |  |  | 62.48(10.57) |  |
| No | 24 | 61.81(10.63) |  |  | 62.49(15.40) |  |
| **Email address** |  |  | .69 |  |  | .91 |
| Yes | 51 | 60.95(9.79) |  |  | 62.35(10.33) |  |
| No | 37 | 61.82(10.62) |  |  | 62.65(14.10) |  |
| **Seriousness on video gaming** |  |  | .52 |  |  | .45 |
| Non-casual player | 58 | 61.82(10.71) |  |  | 63.18(11.39) |  |
| Casual player (Casual/ Very casual) | 30 | 60.34(8.89) |  |  | 61.11(13.15) |  |
| **Frequency on video gaming** |  |  | .11 |  |  | .23 |
| Daily | 22 | 64.29(10.74) |  |  | 65.15(11.57) |  |
| Less than daily | 66 | 60.32(9.76) |  |  | 61.59(12.08) |  |
| **Proportion of spare time spent on video gaming** |  |  | .39 |  |  | .06 |
| Use more of one's spare time | 37 | 62.41(9.40) |  |  | 65.34(12.45) |  |
| Use less of one's spare time | 51 | 60.52(10.59) |  |  | 60.40(11.31) |  |

**P*<.05
